# Anabolic–androgenic steroid abuse: psychiatric manifestations and treatment

**DOI:** 10.1192/j.eurpsy.2023.1387

**Published:** 2023-07-19

**Authors:** M. Remelhe, P. Barbosa, R. Silva

**Affiliations:** Centro Hospitalar Vila Nova de Gaia/Espinho, Vila Nova de Gaia, Portugal

## Abstract

**Introduction:**

Anabolic-androgenic steroids (AAS) are synthetic derivatives of steroids which are frequently utilized in order to increase muscle mass and strength. Estimates of AAS abuse vary according to different studies. However, a global lifetime prevalence of 3.3% has been reported. The increasing illicit use of AAS over the years has raised serious public health concerns.

**Objectives:**

To report a clinical case of AAS abuse and review the psychopathology associated with AAS abuse and its treatment.

**Methods:**

The authors report a clinical case and conducted a non-systematic review on the Pubmed database with the terms “anabolic-androgenic steroids”, “abuse” and “psychiatric manifestations”.

**Results:**

A 25-year-old male patient with history of body dysmorphic disorder was admitted to the emergency services following severe psychomotor agitation and verbal and physical aggression towards his family members. He explained he had begun using AAS years prior in order to improve his physical appearance. Recently he had been administering AAS injections on a more regular basis and restricting his food and water intake. His sleep-wake cycle was disrupted and he began experiencing increasing anxiety and persecutory ideation. Additionally, increased aggression was reported and numerous conflicts with his family members took place in the weeks previous to his psychiatric observation. AAS abuse has been associated with psychiatric symptoms such as aggression and violence, mania and less commonly psychosis and suicidal ideation. Moreover, its prolonged abuse can lead to symptoms of dependence and withdrawal following suspension. A biphasic model of steroid dependence has been described. First there is a brief hyperadrenergic state with opoid-like withdrawal symptoms which is then followed by a prolonged period of depression and craving. During this phase symptoms such as fatigue, muscle and joint pain, insomnia, anxiety and depression may occur. There are no established guidelines for treatment. Acute care of agitation should follow a similar course as the one utilized in other forms of substance induced-agitation: firstly, the least invasive interventions should be implemented. If medication is required, haloperidol has been reported to be effective, although evidence is scarce. Benzodiazepines may be considered, although its use with AAS has not been reported. In regards to long term care, AAS discontinuation is vital in conjunction with proper management of withdrawal symptoms. If a patient presents symptoms of opioid-like withdrawal, treatment with clonidine may be initiated. The use of medically prescribed steroids has been suggested in order to alleviate withdrawal symptoms. Other treatment options such as human chorionic gonadotrophin and clomiphene have also been proposed.

**Conclusions:**

AAS abuse is a serious public health concern. Clinicians should be aware of its serious psychiatric effects and possible treatment courses.

**Disclosure of Interest:**

None Declared

